# Neural Plasticity in a French Horn Player with Bilateral Amelia

**DOI:** 10.1155/2021/4570135

**Published:** 2021-07-30

**Authors:** Daniel S. Scholz, Marcus Heldmann, Bahram Mohammadi, Thomas F. Münte, Eckart Altenmüller

**Affiliations:** ^1^Institute of Music Physiology and Musicians' Medicine, University of Music, Drama and Media, Hannover, Germany; ^2^Department of Neurology, University of Lübeck, Lübeck, Germany; ^3^International Neuroscience Institute, Hannover, Germany

## Abstract

Precise control of movement and timing play a key role in musical performance. This motor skill requires coordination across multiple joints, muscles, and limbs, which is acquired through extensive musical training from childhood on. Thus, making music can be a strong driver for neuroplasticity. We here present the rare case of a professional french horn player with a congenital bilateral amelia of the upper limbs. We were able to show a unique cerebral and cerebellar somatotopic representation of his toe and feet, that do not follow the characteristic patterns of contralateral cortical and ipsilateral cerebellar layout. Although being a professional horn player who trained his embouchure muscles, including tongue, pharyngeal, and facial muscle usage excessively, there were no obvious signs for an expanded somatosensory representation in this part of the classic homunculus. Compared to the literature and in contrast to control subjects, the musicians' foot movement-related activations occurred in cerebellar areas that are typically more related to hand than to foot activation.

## 1. Introduction

Here, we present the case of an 18 year-old male professional French horn player born with bilateral amelia of the upper extremities (called “the musician” further on). He is pushing the valves of his instrument with the toes of his dominant left foot. Almost a child prodigy, he practiced music since his early childhood and was studying music at a prestigious conservatory in northern Germany at time of measurement. Due to his musical proficiency and the amount of daily practice, he can be considered a professional musician. Accordingly, the functional organization of the musicians' cortical representation is driven by training his instrument, but also by the demand of using his feet at a skill level that resembles the fine-grained abilities of subjects with normal hand use.

Size and temporal organization of cortical representations is continually shaped by environmental demands and experience throughout the lifespan [1, 2]. And motor skill acquisition promotes human brain plasticity [3]. Especially in musicians, changes in cortical representations are the outcome of years of extensive practice acquiring highly differentiated motor and auditory skills [4–9]. Improvements in auditory perceptual skills [10–14] and sensorimotor learning [15–17] are accompanied by structural changes [18–22] reflecting the outlasting impact of music training on the human brain's plasticity.

The loss or the absence of sensory input is another plasticity driving factor [23–26]. Cortical plasticity within the sensorimotor homunculus [27, 28] occurs if amelia or amputation causes a lack of input and output for the motor and somatosensory cortex [29–32]. For example, Yu et al. [33] reported in two subjects with missing hands that toe tapping is not only associated with activation in the foot primary motor cortex but also with activation in a brain site typically identified as the hand knob area [34]. However, there seem to be differences in the reorganizational principles of cortical plasticity between amputees and persons with congenital amelia. While in amputees, the cortical representations of lost upper or lower extremities can persist over time [32, 35–37], and persons with congenital amelia reveal a different cortical organization of the somatosensory and motor cortex driven by the compensatory overuse of the intact extremities [32]. Some authors argue that this reorganization process is not restricted to spatially neighbored brain areas and accordingly can result in a nonsomatotopical representation within the motor and somatosensory cortex ([38, 39], but see [40]). Similar plasticity driven alterations of the cerebellums' somatotopy [41–43] are reported by Hahamy & Makin [44]. Investigating subjects with congenitally one hand, they are able to show that the deprived-hand region of the cerebellum can represent multiple body parts. Based on their findings, they conclude that like for the cerebrum, the reorganization of cerebellar somatotopical representations is not restricted to the spatial layout of neuroanatomical constraints, but is driven by the environmental demands.

By using functional magnet resonance imaging (fMRI), we are able to show the impact of two plasticity driving factors on the somatotopical organization of cerebrum and cerebellum: the loss of sensory input and output caused by the participant's amelia of the upper limbs and practicing a musical instrument at a professional level.

## 2. Methods

### 2.1. Subjects

#### 2.1.1. Musician

An 18 year-old male professional French horn player born with bilateral amelia of the upper extremities was investigated. He is pushing the valves of his instrument with the toes of his dominant left foot. He started playing French horn at the age of six. He successfully managed all daily living activities with his feet and was capable of 8 toe writing on a special computer keyboard. He could dress and undress with his feet with the help of a small mechanical device. He held a regular highschool degree. He did not show any phantom pain or any other pain related to the different organization of his body and brain. No neurological or psychiatric disorder was present.

#### 2.1.2. Control Subjects

Ten healthy subjects were recorded (age 18-30 years, all male). Before participating in our study, they gave written informed consent.

#### 2.1.3. MRI Data Recording

The musician and three of the control subjects were recorded at a 3-T Siemens Magnetom Scanner (Erlangen, Germany) equipped with a standard head coil. Functional images were recorded using a T2^∗^-weighted whole brain Echo-planar imaging (EPI) sequence (TR 2000 ms, TE 30 ms, flip angle 80°, field-of-view (FOV) 192 mm, matrix 64 × 64, 34 slices, slice thickness 3 mm, interslice gap 0.75 mm). Data of two control subjects were acquired at a 3T Philipps Ingenia scanner with an 8 channel head coil using a T2^∗^ EPI-Sequence and the following parameters: matrix 64 × 64, 3 mm isovoxel, TR 2000 ms, TE 25 ms, flip angle 80°, and 43 slices. Finally, five control subjects were recorded in a 3T Siemens Skyra Scanner using a 64 channel head coil. For the functional recordings, a T2^∗^ EPI sequence (TR 2000 ms, TE 30 ms, flip angle 70°, 54 slices, 3 mm isovoxel, FOV 192 mm, matrix 64 × 64) with simultaneous multislice recording (slice acceleration factor: 2) was used. All functional data were recorded aligned parallel to the AC-PC line. The plantar flexion task comprised 80 volumes, and all sensory stimulation tasks 160 volumes. In all participants, T1-weighted high-resolution data were acquired using a 3D-MPRAGE sequence with a resolution of 1 mm isovoxel (Siemens Scanners matrix 192 × 256, Philipps Scanner 240 × 240). Each subjects' head was fixed during the entire measurement to avoid head movements.

#### 2.1.4. Experimental Design

The functional recording comprised a motor and a sensory stimulation task. The motor task comprised a plantar flexion task of the right and the left foot. For each foot, one functional run was performed comprising four blocks of alternating task and rest conditions, each lasting twenty seconds. The frequency of the foot movements was practiced outside of the scanner to assure a highly similar frequency across the subjects. The sensitive stimulation task involved the 1st, 3rd, and 5th toe of each foot. For each toe of each foot, one run was performed. Within a run, the toe was stimulated by tipping (frequency 1 Hz) with the butt end of a wooden stick (1 mm diameter) at the toe bottom. Like for the plantar flexion task, each run comprised four blocks of alternating task and rest conditions, each lasting twenty seconds. The stimulation was performed by TM and DSS.

#### 2.1.5. Preprocessing and Statistical Analysis

Preprocessing and statistical analysis was performed using Statistical Parametric Mapping (SPM) 12 (version 7219) (https://www.fil.ion.ucl.ac.uk/spm/software/) running under Matlab 2017b (mathworks.com) [45–47]. Preprocessing of the functional data comprised correction for slice acquisition time by phase-shifting each volume's slice with reference to the middle slice. In order to remove movement artifacts, volumes of each subject were realigned. Images were normalized to Montreal Neurological Institute (MNI) space by first coregistering the T1 to the mean EPI image. In a next step, T1 images were segmented into grey and white matter and normalized to MNI space. The resulting transformation matrices were used to transform functional data to MNI space. In order to get comparable statistics, all functional data were resliced to 3 mm isovoxel. Subsequently, functional data were smoothed with a Gaussian kernel of 5 mm full-width half-maximum. A filter width of 128 s was used for temporal high pass filtering.

At the first level, two general linear models (GLM) were calculated per subject, one for the plantar flexion, one for the sensory stimulation task. The plantar flexion task comprised two runs, one each for the left and right foot resulting in a model with two task (left/right foot) and six movement-related regressors. The sensory stimulation task-related GLM comprised six runs with two tasks (summarizing the activation of 1st, 3rd, and 5^th^ toe per foot) and six movement-related regressors. For the musician, we report the resulting first-level analysis with *p* = 0.05 (family-wise error (FWE)). The control subjects are tested for activations different from zero at a group level by applying a one-sample *t*-test per condition. All group results were reported at with cluster level corrected *q* = 0.05 (FWE, *p* = 0.001 cluster defining threshold). In addition, cerebellar activations are investigated restricting the GLM to the cerebellum as defined by the mask in MNI space provided by the SUIT toolbox [48]. Please note the varying statistical thresholds for the musicians' first level results (plantar flexion task: *p* = 0.05 (FWE), sensory stimulation: *q* = 0.05 (FWE, cluster defining threshold *p* = 0.001)) and the one sample *t*-test of the control group (plantar flexion task *q* = 0.05 (FWE, *p* = 0.001 cluster defining threshold) and sensory stimulation *q* = 0.05 (FWE, *p* = 0.005 cluster defining threshold)).

**Figure 1:**
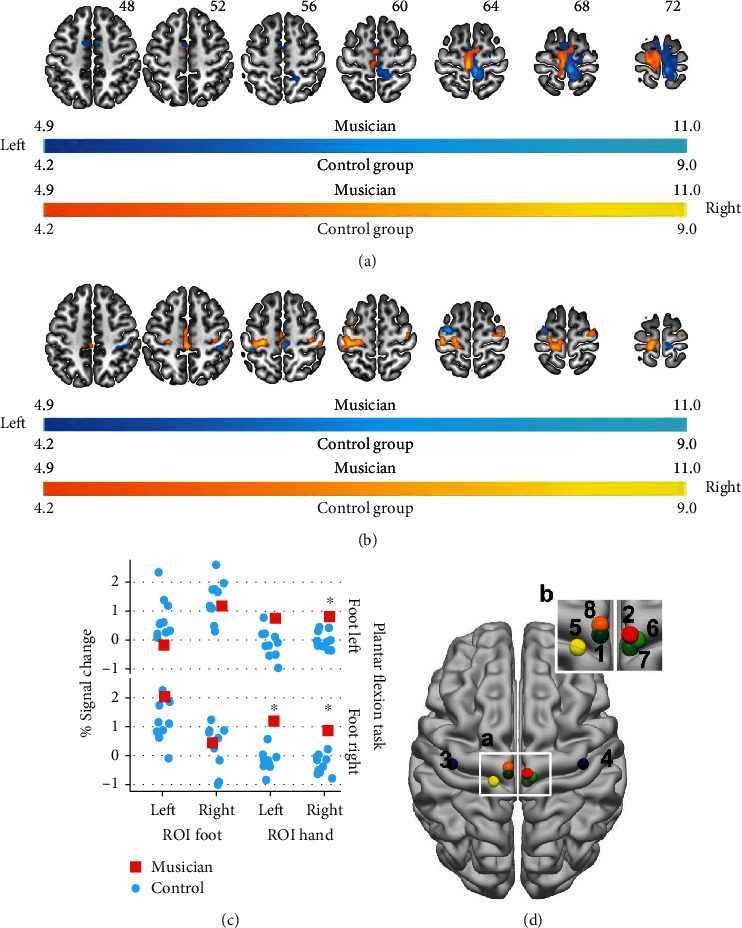
Cortical activations related to the plantar flexion task. (a) displays the musicians' activation and (b) the group mean. Images are displayed in neurological conventions. Activation related to the left foot is displayed in bluish-green and activation related to the right foot in reddish-yellow. The musicians' results are corrected for multiple comparisons *p* = 0.05 (FWE), and the group level analysis is corrected using *q* = 0.05 (FWEc, cluster defining threshold = *p* = 0.005). In (c), the mean % signal change for the foot and the hand ROIs are displayed. Asterisk indicates a significant difference (*p* = 0.05) for the plantar flexion task-related activation in the hand ROIs between the musician and the control group. (d) ROI location and peak activation of the plantar flexion task: 1-4: ROIs defined by Buckner [42]: foot left hemisphere (1), foot right hemisphere (2), hand left hemisphere (3), and hand right hemisphere (4); 5-8: peak activations plantar flexion task: musician task right (5), musician task left (6), group task left (7), and group task right (8). (b) is a magnified view of (a). Also, see Table 1 in the supplementary materials.

**Figure 2:**
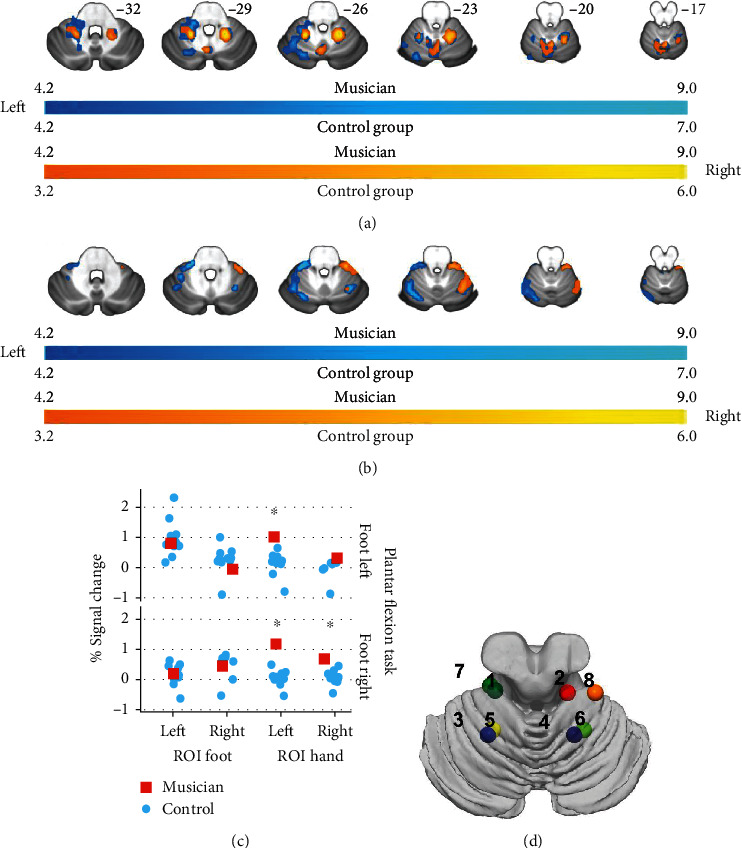
Cerebellar activations related to the plantar flexion task. (a) displays the musicians' activation and (b) the group mean. Images are displayed in neurological conventions. Activation related to the left foot is displayed in bluish-green and activation related to the right foot in reddish-yellow. The musicians' results are corrected for multiple comparisons *p* = 0.05 (FWE), and the group level analysis is corrected using *q* = 0.05 (FWEc, cluster defining threshold = *p* = 0.001). In (c), the mean % signal change for the foot and the hand ROIs is displayed. Asterisks indicate a significant difference (*p* = 0.05) for the plantar flexion task-related activation in the hand ROIs between the musician and the control group. (d) ROI location and peak activation of the plantar flexion task: 1-4: ROIs defined by Buckner [42]: foot left hemisphere (1), foot right hemisphere (2), hand left hemisphere (3), and hand right hemisphere (4); 5-8: peak activations plantar flexion task: musician task right (5), musician task left (6), group task left (7), and group task right (8). Also, see Table 2 in the supplementary materials.

**Figure 3:**
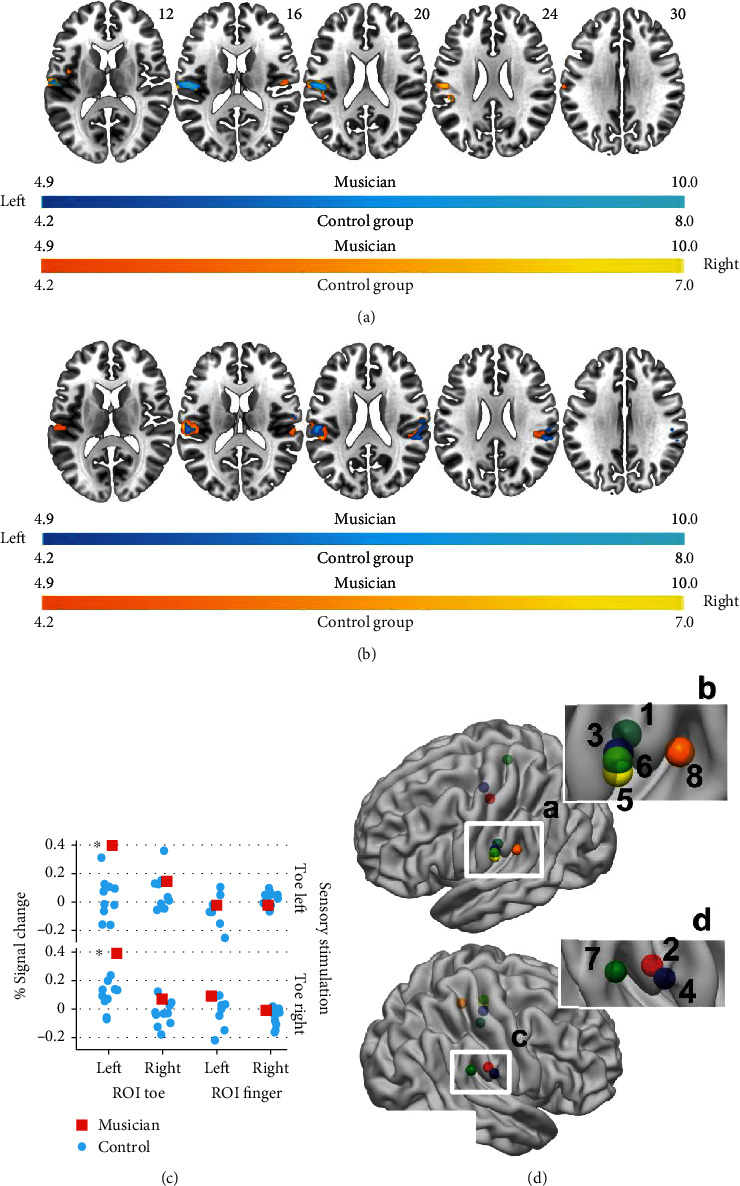
Cortical activations related to the sensory stimulation task. (a) displays the musicians' activation and (b) the group mean. Images are displayed in neurological conventions. Activation related to the left toe is displayed in bluish-green and activation related to the right toe in reddish-yellow. The musicians' results are corrected for multiple comparisons *p* = 0.05 (FWE), and the group level analysis is corrected using *q* = 0.05 (FWEc, cluster defining threshold *p* = 0.001). In (c), the mean % signal change for the toe and the finger ROIs is displayed. Asterisks indicate a significant difference (*p* = 0.05) for the sensory stimulation related activation in the left toe ROIs between the musician and the control group. (d) ROI location and peak activation of the plantar flexion task: 1-4: ROIs defined by Buckner [42]: foot left hemisphere (1), foot right hemisphere (2), hand left hemisphere (3), and hand right hemisphere (4); 5-8: peak activations plantar flexion task: musician task right (5), musician task left (6), group task left (7), and group task right (8). (b) is a magnified view of (a), and (d) is a magnified view of (c). Also, see Table 3 in the supplementary materials.

Region of interest (ROI) analysis: for each functional task, a ROI analysis was performed. For the plantar flexion task, spheres with a radius of 10 (cortex) or 6 (cerebellum) mm centered around the coordinates described by Buckner [42] for the foot and hand-related functional activation were defined. To determine the activation related to the sensory stimulation, 10 mm ROIs as described by Ruben et al. [49] for toe and finger activation located in the secondary somatosensory cortex are used. For the cerebellum, we used the cerebellar ROIs from the plantar flexion task, please see Figures 1–3 for the ROIs' localization. In order to reveal differences between the musician and healthy controls, mean percent signal changes per ROI and condition were subjected to a Bayesian test for single case analysis [50]. A significant test indicates that the musicians' percent signal change in the corresponding ROI is significantly different from the group of healthy controls. Significance level was set to *p* = 0.05.

## 3. Results

The analysis of the plantar flexion task revealed for the control group the expected activations contralateral to the activated foot in the paracentral lobe (Figure 1(b)). In contrast, for the musician, a more left lateralized activation pattern is observed; although, cortical activations contralateral to the foot used can also be seen (Figure 1(a)). The analysis of the extracted percent signal change indicated for the foot-related ROIs (Figure 1(d): spheres 1 and 2), that the musician is not significantly different from the control group. In contrast, the analysis of the hand-related ROIs (Figure 1(d): spheres 3 and 4) revealed that the musicians' activation is significantly different from the control subjects (plantar flexion left: ROI hand left *p* = 0.056 [n.s.], ROI hand right *p* = 0.015; plantar flexion right: ROI hand left *p* = 0.002, ROI hand right *p* = 0.004).

Restricting the analysis of the plantar flexion task to the cerebellum revealed for the musician bilateral activation for the plantar flexion of the left and right foot. Moreover, peak activation (see Table 2, supplementary material) occurred contralateral to the motor activity in cerebellar sites that are described by Buckner to be related to hand activity. The corresponding analysis of the control group resulted in activities ipsilateral to the activated foot and occurring at brain sites that are known to represent the motoric foot activity.

The analysis of the extracted percent signal change for the foot-related cerebellar ROIs (Figure 2(d): spheres 1 and 2) revealed a pattern of results similar to the analysis of the cortical ROIs: no significant differences for the foot-related ROIs (Figure 2(d): spheres 1 and 2), but significant differences between musician and control group in three out of four ROIs (plantar flexion left: ROI hand left *p* = 0.028, ROI hand right *p* = 0.23 [n.s.]; plantar flexion right: ROI hand left *p* = 0.001, ROI hand right *p* = 0.02).

**Figure 4:**
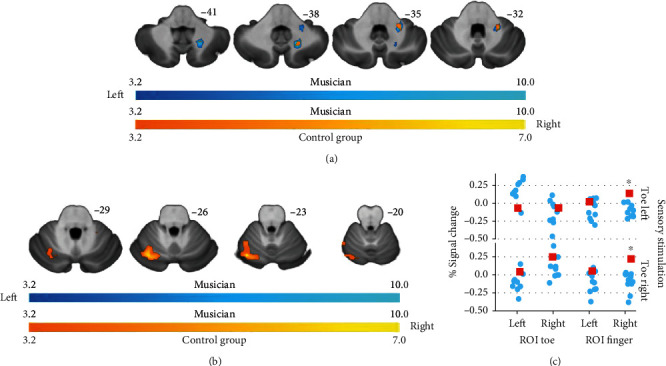
Cerebellar activations related to the sensory stimulation task. (a) displays the musicians' activation and (b) the group mean. Images are displayed in neurological conventions. Activation related to the left foot is displayed in bluish-green and activation related to the right foot in reddish-yellow. The musicians' results are corrected for multiple comparisons *q* = 0.05 (false discovery rate (FDR)c, cluster defining threshold *p* = 0.001), and the group level analysis is corrected using *q* = 0.05 (FDRc, cluster defining threshold *p* = 0.005). In (c), the mean % signal change for the foot and the hand ROIs is displayed. Asterisks indicate a significant difference (*p* = 0.05) for the sensory stimulation task-related activation in the right hand ROIs between the musician and the control group for the sensory stimulation of the left and right toe. Also, see Table 4 in the supplementary materials.

The sensory stimulation task revealed at the cortical level for the musician activations in the left secondary somatosensory cortex. The only activation that is found contralateral to the stimulated toe is a significant cluster in the right Rolandic Operculum when stimulating the left toe. In contrast, the control group analysis resulted in the expected peak activations contralateral to the stimulation site. The ROI analysis revealed for the left toe ROI only a significant difference between musician and controls (stimulation toe left *p* = 0.018, toe right *p* = 0.009). Restricting the analysis of the sensory stimulation task to the cerebellum (Figure 4) revealed for the musician an overlap of activation to the stimulation of the left and right toe in the lobule VIII. The analysis of the control group resulted in a significant activation of the left lobule VI when stimulating the ipsilateral toe. The stimulation of the right toe did not cause a significant cerebellar activation. The reported overlap is also reflected in the ROI analysis showing a significant difference between musician and controls for the analysis of the cerebellar hand-related ROI (stimulation left toe *p* = 0.01, right toe *p* = 0.02).

## 4. Discussion

In this rare case of a professional French horn player with bilateral amelia of the upper limbs, we are able to show a unique cerebral and cerebellar somatotopic representation of his toes and feet, that does not follow the characteristic pattern of contralateral cortical and ipsilateral cerebellar layout.

Compared to the literature [41–44, 49] and in contrast to control subjects, the musicians' foot movement-related activations occur in cerebellar areas that are typically more related to the hand than to foot activation. This topological movement away from areas that usually represent the feet towards areas representing the hands is, at the cortical level, already described for an amelic man without hands [31]. The reported bilateral activation for the right foot and toe movement deviates from the usually reported pattern of cerebellar activation ipsilateral to the active hand or foot. The analysis of the activation-based ROIs confirmed the peculiarity of the musicians' cortical and cerebellar functional organization. Instead of the expected cortical contralateral and a cerebellar ipsilateral representation of the activated foot, a left-sided cortical and a right-sided cerebellar dominance were observed, irrespective of the side of the activated foot. The obviously increased sensitivity of the musician of both feet in comparison to the healthy control group is in line with a recent report by Dempsey-Jones [51], which also describes an increased sensitivity of extreme foot users. Although we would like to argue that this could be a sign of plasticity and reflects the musicians' ability to recruit a broader neural basis to execute more fine-grained foot movements, one has to take into account that faster foot movements can also result in bilateral activation of the cerebellum [52, 53]. At the cortical level, the observed contralateral representation of his right foot is comparable to the foot representations in amputees reported by Yu et al. [33, 54], showing that movement-related activation at the primary foot motor cortex is accompanied by extended activation nearby the contralateral hand knob. This similarity is interesting to note, since in contrast to the musician born without arms, the participants reported by Yu et al. [33, 54] are all secondary arm amputees, indicating that irrespective of the causes for a missing limb and the age of onset of cortical reorganization, compensatory use can result in similar patterns of cortical plasticity.

Based on the known effects of practicing in musicians, we assumed that the somatotopic representation of the dominant left foot of the musician would be affected most by his excessive motoric training. Of course, with the methodology applied in this study (fMRI), we are not able to distinguish whether the different functional organizations of the musician's brain is caused by long-term training of the instrument or the mere absence of the upper limbs right from birth on. Unexpectedly, we observed in the cerebellum right lateralized activations consisting of two activation maxima. According to Buckner [42], Buckner [41] and Grodd [55], these activations occur locally where hand and face/lips/tongue are usually represented. However, the violation of the organizational principle of ipsilateral limb representation in the cerebellum was to our knowledge not reported somewhere else before. Usually, in brass players, valve usage causes cerebellar activation ipsilateral to the hand used [56]. Even in brass players with reported changes in their somatosensory system due to embouchure dystonia, a disorder believed to be related to maladaptive brain plasticity, no such activation flip between cerebellar hemispheres occurred [57]. Since we controlled carefully for coactivations of the unused foot during the task, we would like to rule out that these activations are caused by additional movement of the right foot. The cortical activations caused by the motor activation of the left foot confirmed the impression of a highly uncommon somatotopic representation, occurring at the ipsi- and contralateral pre- and postcentral gyrus and overlapping with right foot movement-related activations. This overlap of right foot movement resembles the pattern of overlap observed in the cerebellum as well.

Hahamy and colleagues (2018, 2019) recently argued for a reorganization of body part representations that is not limited to somatotopic constraints (see also [58]). In line with previous literature, they demonstrated that such reorganization not only occurs at cortical but also at cerebellar level [44]. However, neither they nor others reported a plasticity driven flip in the hemispheric assignment. In sum, we are able to present a rare case of extensive reorganization of the brain where the combination of innate amelia and excessive musical practicing reveals the potential power of neuroplasticity.

## Data Availability

The dataset supporting the conclusions of this article is included within the article.

## Supplementary Materials

Supplementary Materials Supplementary materials consist of Table 1: cortical activations related to the plantar flexion task. Table 2: cerebellar activations related to the plantar flexion task. Table 3: cortical activations related to the sensory stimulation task. Table 4: cerebellar activations related to the sensory stimulation task.
