# Supplementary material for: Neural Plasticity in a French Horn Player with Bilateral Amelia
Source: Neural Plast. 2021 Jul 30;2021:4570135. doi: 10.1155/2021/4570135 (PMC8349270; doi:10.1155/2021/4570135)
Supplement: Supplementary Materials — consist of Table 1: cortical activations related to the plantar flexion task. Table 2: cerebellar activations related to the plantar flexion task. Table 3: cortical activations related to the sensory stimulation task. Table 4: cerebellar activations related to the sensory stimulation task. [file 4570135.f1.docx]

***Table 1:***

| **Musician: foot left FWE; *p*=0.05, k=10** | | | | |
| --- | --- | --- | --- | --- |
| Hemisphere | AAL label | Cluster Size | max T | Coordinates |
| left | Precentral gyrus | 24 | 9.23 | -30 -10 67 |
| right | Lobule VI | 26 | 7.64 | 27 -49 -26 |
| right | Paracentral Lobule | 13 | 7.35 | 9 -28 73 |
| left | Lobule IV, V | 18 | 6.46 | -21 -49 -23 |
| left | Paracentral Lobule | 19 | 6.26 | 0 -28 58 |
| left | SMA |  | 5.19 | 0 -19 55 |
| left | Postcentral gyrus | 10 | 6.19 | -39 -34 58 |
| right | Postcentral gyrus | 22 | 6.09 | 42 -31 52 |
|  |  |  | 5.85 | 33 -31 46 |
| **Musician: foot right FWE; *p*=0.05, k=10** | | | | |
| left | Precentral gyrus | 205 | 11.24 | -30 -10 67 |
|  |  |  | 8.84 | -27 -25 55 |
| left | Paracentral Lobule |  | 8.34 | -15 -31 70 |
| right | Lobule IV, V | 68 | 9.31 | 24 -46 -23 |
| left | Lobule IV, V | 40 | 8.61 | -18 -49 -23 |
| left | Paracentral Lobule | 51 | 7.90 | 0 -28 55 |
| left | SMA |  | 5.92 | -3 -10 52 |
| right | Precentral gyrus | 44 | 7.24 | 36 -22 55 |
|  |  |  | 6.37 | 30 -13 70 |
|  |  |  | 5.50 | 36 -7 64 |
| left | Rolandic Operculum | 12 | 7.13 | -45 -22 19 |
| right | Postcentral gyrus | 19 | 6.53 | 48 -28 52 |
| right |  |  | 5.59 | 42 -34 55 |
| right | Lobule IV, V of vermis | 46 | 6.18 | 3 -61 -11 |
|  | Lobule VI of vermis |  | 5.94 | 0 -64 -23 |
| left | Lobule IV, V |  | 5.85 | -6 -58 -11 |
| right | Rolandic Operculum | 10 | 5.94 | 51 2 4 |

| **Control group: foot left FWEc; *p*=0.01, k=47** | | | | |
| --- | --- | --- | --- | --- |
| Hemisphere | AAL label | Cluster Size | max T | Coordinates |
| right | Paracentral Lobule | 418 | 14.09 | 6 -31 67 |
|  |  |  | 8.97 | 9 -43 67 |
| right | Precentral gyrus |  | 7.81 | 12 -28 76 |
| left | Lobule IV, V of vermis | 314 | 9.23 | -3 -52 -8 |
| left | Lobule IV, V of vermis |  | 8.91 | -3 -46 -14 |
|  |  |  | 8.79 | -3 -61 -8 |
| right | Putamen | 54 | 8.41 | 21 5 7 |
| left | Putamen | 47 | 6.73 | -21 5 7 |
| left | Caudate Nucleus |  | 6.19 | -18 8 16 |
| right | Paracentral Lobule |  | 4.93 | -18 -1 22 |
| **Control group: foot right FWEc; *p*=0.01, k=47** | | | | |
| left | Paracentral Lobule | 189 | 12.03 | -15 -22 73 |
|  |  |  | 10.56 | -3 -22 67 |
|  |  |  | 7.99 | -6 -22 76 |
| left | Putamen | 54 | 9.53 | -30 -4 7 |
| left | Pallidum |  | 7.16 | -24 -7 1 |
|  | Lobule IV, V of vermis | 61 | 8.46 | 0 -49 -2 |
| right | Lobule IV, V |  | 5.95 | 9 -49 -8 |
|  | Lobule III of vermis |  | 5.17 | 3 -43 -11 |
| left | Rolandic Operculum | 49 | 7.54 | -45 -28 19 |
| left | Superior temporal gyrus |  | 7.00 | -54 -31 19 |
| left | SupraMarginal gyrus |  | 6.65 | -57 -22 16 |

***Table 2:***

| **Musician: Cerebellum - foot left FWE; *p=*0.01, k=10** | | | | |
| --- | --- | --- | --- | --- |
| Hemisphere | AAL label | Cluster Size | max T | Coordinates |
| right | Lobule VI | 59 | 11.16 | 21 -50 -26 |
| left | Lobule IV, V | 167 | 10.66 | -18 -50 -26 |
| left | Lobule IV, V |  | 8.35 | -18 -39 -31 |
| left | Crus I of cerebellar hemisphere |  | 7.63 | -21 -74 -25 |
| left | Lobule VII of vermis | 69 | 8.39 | -3 -71 -22 |
| right | Lobule VI of vermis |  | 7.41 | 3 -62 -18 |
| left | Lobule IV, V |  | 7.22 | -6 -59 -18 |
| **Musician: Cerebellum - foot right FWE; *p=*0.01, k=10** | | | | |
| left | Lobule IV, V | 37 | 9.84 | -18 -50 -26 |
| left | Lobule VI |  | 5.48 | -30 -45 -30 |
| right | Lobule IV, V | 66 | 9.72 | 18 -47 -27 |
| right | Lobule VI |  | 8.06 | 15 -56 -22 |
| right | Lobule VI of vermis | 54 | 8.21 | 3 -62 -18 |
| left | Lobule VII of vermis |  | 6.87 | -3 -69 -29 |
| left | Lobule IV, V |  | 6.33 | -9 -59 -18 |

| **Control group: Cerebellum - foot left FWEc; *p=*0.001, k=31** | | | | |
| --- | --- | --- | --- | --- |
| Hemisphere | AAL label | Cluster Size | max T | Coordinates |
| left | Lobule IV, V | 315 | 9.67 | -19 -31 -30 |
| left |  |  | 9.06 | -4 -46 -9 |
| left |  |  | 7.73 | -25 -34 -24 |
| right | Lobule VI | 31 | 7.63 | 29 -61 -27 |
| **Control group: Cerebellum - foot right FWEc; *p=*0.005, k=90** | | | | |
| right | Lobule IV, V | 90 | 5.37 | 26 -34 -24 |
| right |  |  | 4.96 | 17 -34 -21 |
| right | Lobule VI |  | 4.89 | 35 -40 -27 |

***Table 3:***

| **Musician: toe left FWE; *p*=0.05, k=10** | | | | |
| --- | --- | --- | --- | --- |
| Hemisphere | AAL label | Cluster Size | max T | Coordinates |
| left | Postcentral gyrus | 350 | 13.47 | -51 -19 19 |
| left | SupraMarginal gyrus |  | 11.51 | -57 -25 37 |
| left | Parietal_inferior gyrus |  | 9.94 | -51 -28 46 |
| left | Precentral gyrus | 75 | 10.92 | -36 -7 64 |
| left | Middle frontal gyrus |  | 7.21 | -30 8 64 |
| left | Precentral gyrus | 49 | 9.03 | -54 -1 43 |
| right | Lobule VI of cerebellar hemisphere | 12 | 8.48 | 18 -70 -23 |
| left | Rolandic_Operculum | 28 | 7.55 | -45 -4 7 |
| left | Middle cingulate gyrus | 17 | 6.87 | -3 -25 49 |
| right | Rolandic_Operculum | 17 | 6.13 | 51 -16 16 |
| **Musician: toe right FWE; *p*=0.05, k=10** | | | | |
| left | Rolandic_Operculum | 99 | 9.79 | -51 -19 16 |
| left | Superior temporal gyrus |  | 6.04 | -45 -34 22 |
| left | Parietal_Inferior gyrus | 60 | 7.87 | -54 -25 37 |
| left | Parietal_Inferior gyrus |  | 7.40 | -51 -28 46 |
| left | Precentral gyrus | 32 | 7.33 | -36 -4 64 |
| left | Postcentral gyrus | 14 | 5.81 | -39 -37 55 |

| **Control group: toe left FWEc; *p*=0.001, k=34** | | | | |
| --- | --- | --- | --- | --- |
| Hemisphere | AAL label | Cluster Size | max T | Coordinates |
| right | SupraMarginal gyrus | 49 | 7.91 | 57 -37 28 |
| right | Postcentral gyrus |  | 5.86 | 57 -13 22 |
| right | SupraMarginal gyrus |  | 5.64 | 60 -22 22 |
| left | SupraMarginal gyrus | 34 | 7.10 | -54 -25 19 |
| **Control group: toe right FWEc; *p*=0.001, k=43** | | | | |
| left | Superior temporal gyrus | 72 | 8.37 | -48 -34 19 |
| left | SupraMarginal gyrus |  | 7.44 | -60 -22 16 |
| left | Rolandic_Operculum |  | 6.51 | -48 -25 16 |
| right | SupraMarginal gyrus | 43 | 5.93 | 57 -25 19 |
| right | Rolandic_Operculum |  | 5.49 | 45 -31 22 |
| right | Postcentral gyrus |  | 5.35 | 60 -13 19 |

***Table 4:***

| **Musician: Cerebellum - toe left FDRc; *p*=0.001, k=21** | | | | |
| --- | --- | --- | --- | --- |
| Hemisphere | AAL label | Cluster Size | max T | Coordinates |
| right | Lobule VIII of cerebellar hemisphere | 21 | 8.35 | 19 -61 -37 |
| right | Cerebelum_10_R | 24 | 6.55 | 25 -42 -37 |
| **Musician: Cerebellum - toe right FDRc; *p*=0.001, k=10** | | | | |
| right | Lobule VI of cerebellar hemisphere | 7 | 5.13 | 25 -43 -33 |
| right | Lobule VIII of cerebellar hemisphere | 10 | 4.92 | 19 -61 -37 |

| **Control group: Cerebellum - toe left FDRc; *p*=0.005, k=62** | | | | |
| --- | --- | --- | --- | --- |
| Hemisphere | AAL label | Cluster Size | max T | Coordinates |
| left | Lobule VI of cerebellar hemisphere | 62 | 9.90 | -28 -70 -24 |
|  |  |  | 4.22 | -28 -58 -24 |
